# Origin of Emotion Effects on ERP Correlates of Emotional Word Processing: The Emotion Duality Approach

**DOI:** 10.1371/journal.pone.0126129

**Published:** 2015-05-08

**Authors:** Kamil Konrad Imbir, Maria Teresa Jarymowicz, Tomasz Spustek, Rafał Kuś, Jarosław Żygierewicz

**Affiliations:** 1 Faculty of Applied Social Sciences, The Maria Grzegorzewska University, Warsaw, Poland; 2 Faculty of Psychology, University of Warsaw, Warsaw, Poland; 3 Faculty of Physics, University of Warsaw, Warsaw, Poland; Zhejiang Key Laborotory for Research in Assesment of Cognitive Impairments, CHINA

## Abstract

We distinguish two evaluative systems which evoke automatic and reflective emotions. Automatic emotions are direct reactions to stimuli whereas reflective emotions are always based on verbalized (and often abstract) criteria of evaluation. We conducted an electroencephalography (EEG) study in which 25 women were required to read and respond to emotional words which engaged either the automatic or reflective system. Stimulus words were emotional (positive or negative) and neutral. We found an effect of valence on an early response with dipolar fronto-occipital topography; positive words evoked a higher amplitude response than negative words. We also found that topographically specific differences in the amplitude of the late positive complex were related to the system involved in processing. Emotional stimuli engaging the automatic system were associated with significantly higher amplitudes in the left-parietal region; the response to neutral words was similar regardless of the system engaged. A different pattern of effects was observed in the central region, neutral stimuli engaging the reflective system evoked a higher amplitudes response whereas there was no system effect for emotional stimuli. These differences could not be reduced to effects of differences between the arousing properties and concreteness of the words used as stimuli.

## Introduction

Our approach to emotions draws on theories about the duality of the human mind [1–3]. Following appraisal theories [4, 5] we seek to distinguish between emotional reactions to stimuli which are automatic, direct and uncontrolled and those based on deliberation, reflection and appraisal [6]. This study is a part of a program of research on brain mechanisms underlying automatic and reflective emotions [7–11]. Research on the psychology of emotion already recognizes the existence of an Automatic Evaluative System (AES) [7, 9]; however the Reflective Evaluative System (RES) has not been the subject of special attention, although Gazzaniga ([12], p. 218) has pointed out that “We are people, not brains. We are that abstraction that occurs when a mind, which emerges from a brain, interacts with the brain.” This describes what happens when an individual is engaged in deliberation, for example from the AES perspective reading statistics in a yearbook is a very unemotional, boring activity; however if the RES is activated and the reader deliberates or reflects on the material associations with population size and its growth and food production in Africa may be evoked leading to conclusions that there is no food for everybody, which elicit intensive affective reactions. We believe that applying duality of mind theory to emotions can contribute to our understanding of how emotions influence cognitive processing, for example the processing of emotional words.

### Taxonomy of Human Emotions

Our model, consisting of two evaluative systems [6], is based on Zajonc’s insight that “(some) preferences need no inferences” ([13]; Fig 5 p. 170), but posits that some inferences lead to emotions, choices or decisions. The sequential relationship between affect and cognition—both components of emotion can flow in either direction: (1) affect-cognition or (2) cognition—affect. In fact, in the first case the sequence is: implicit cognition—primary affect—(sometimes) explicit recognition of one’s own emotional state. Sequences of the second type are much more complex. Explicit cognition can be influenced by primary affect, but sometimes reflective information processing is relatively independent of earlier automatic processing, and the sequence runs: explicit cognition—activation of reflective system-specific evaluation criteria—deliberative appraisal—secondary affect.

All stimulus evaluation involves assessing the stimulus against a set of criteria. In the case of the AES, in which processing is automatic [14], these criteria are non-verbal. The so called biological value seems to be fundamental criterion related to preserving life in changing environment [15] This is as well an excellent example of a non-verbal AES criterion. The RES operates on the basis of the conceptual (often abstract) evaluation criteria and involves reasoning about an object or subject. Deliberative appraisal depends on the existence of verbal criteria of evaluation [16]. The RES is therefore only involved in stimulus evaluation and the consequent regulation of behavior only if appropriate criteria of evaluation exist; in the absence of such verbalizable standards humans are insensitive to certain stimulus features and attributes. Of course RES criteria are often based on non-verbal AES criteria or values; the biological value relating to preservation of life [15] leads directly and indirectly to higher-level values.

We investigated the cognitive outcomes of automatic and reflective emotional processing to test our model [6]. The behavioral data suggested that factors such as the origin of the emotion (which reflects the distinction between the AES and RES) modulate attention [17], broadening it in response to presentation of stimuli with reflective origin or making it more difficult to maintain cognitive control [18] after presentation of stimuli with automatic origin (both words and sentences). These behavioral data prompted us to investigate the neural correlates of automatic and reflective processing of emotional words.

### Neural Correlates of Word Processing

There is considerable evidence that various stages of processing of emotional words are correlated with amplitude modulation of event related potentials (ERPs) in specific time ranges: (1) around 100 ms post-stimulus [19, 20]; (2) within the first 200 ms period [21]; (3) the P1-N1 time period [22, 23]; (4) the P2 time period [24] and (5) the P3 and later time period [25–27]. In all these studies, the effects were related to the valence of the words although some of the effects were task dependent and varied according to the method of investigation, for example P2 effects are associated with task-relevant perceptual stimuli [28,29]. In the study reported in this paper we focused on two ERPs associated with processing of emotional words, the early posterior negativity (EPN or N2) and the late positive complex or potential (LPC or LPP) [30, 31].

The first observations of emotional modulation of ERPs related to the negative wave (in common average montage) at temporo-occipital electrode sites around 200–400 ms (EPN); responses to emotional stimuli—both positive and negative—were different from responses to emotionally neutral pictures [28] or words [32–36]. It has been suggested that the EPN reflects reflexive visual attention to emotionally charged stimuli which may facilitate visual processing of such stimuli [29]. EPN modulation is reported to occur in simple paradigms (e.g. [32, 35, 36]; for review see [31]) and has been shown to be task-dependent [37]. Modulation of the EPN is only observed when subjects engage in lexico-semantic analysis of presented words. Hinojosa et al. [37] showed that EPN effects disappeared when the task did not require reading of emotionally charged material. Some researchers have failed to observe emotional effects within this time-window (e.g. [29, 38]). It seems that failure to observe EPN modulation is more likely when a more demanding task is used, for example Kaltwasser et al. [29].

The next observation of emotional effects on ERPs related to the LPC—a positive deflection beginning at the time range of the P3 and other later time range components and lasting for several hundred milliseconds. Affective pictures have been shown to increase LPC amplitudes relative to neutral pictures (e.g., [26, 27, 39]); similar effects have been reported for emotional words (e.g. [24, 25, 36, 40]). These effects presumably reflect more elaborate processing of emotional stimuli owing to their motivational significance [26] or evaluation processes required to carry out the task [31]. Some authors (e.g. [24, 32, 39]) found that positive words evoked a larger LPC than neutral or negative words; others reported the opposite pattern of results (e.g. [36, 41–43]), a greater LPC amplitude to negative words than neutral or positive words. This suggests that other factors modulate the relationship between the emotional valence of stimuli and the amplitude of the LPC. Given the characteristics of the LPC [31] these modulatory factors may relate to stimulus processing requirements.

Russell [44] and Bradley and Lang [45] proposed that emotional stimuli effects on cognition could be explained better in terms of two factors: valence and arousal, which can be used to specify a location in two-dimensional affective space [44]. Emotional effects on ERPs were analyzed in terms of valence and arousal; responses to highly arousing and non-arousing stimuli were compared [25–27, 46, 47]. It was reported that LPCs to highly arousing stimuli were of greater amplitude than those to less arousing stimuli, independent of stimulus valence [48], it has been suggested that this effect reflects sustained and elaborated processing of high-arousal stimuli [39] caused by their higher motivational significance [49].

### Aims and Hypotheses

We assume that the involvement of the two systems of evaluation, AES and RES, in the processing of affective words varies according to the origin of the emotions, operationalized as assessment of first impression [50, 51] that stimuli is related to heart vs. mind. The purpose of this study was to identify and characterize differences in ERP amplitudes related to differences in conditions of reading words connected with distinct types of emotions: negative and positive, of automatic or reflective origin. We hypothesized that the amplitude of some ERP components would depend on both the system generating the emotions elicited by a given stimulus and the valence of those stimuli.

The study used a similar design and lexical stimuli [50, 51] to our earlier behavioral studies [17, 18]. Because this was exploratory research on the role of different emotional systems in ERPs [6, 51] we chose to carry out exploratory analyses of ERPs not limited to EPNs and LPCs in Regions of Interest (ROIs). We predicted that using emotional stimuli would generate effects on early (EPN) and late (LPC) ERPs. We expected that valence would only affect the early stages of processing (EPN) [37]. We hypothesized that the complexity of the underlying appraisal (automatic vs. reflective) would be reflected in late ERP components (LPC).

## Materials and Methods

### Participants

The participants were native Polish-speaking students of the Faculty of Psychology, University of Warsaw (*N* = 25), aged from 19 to 23 years (*M* = 20.4, *SD* = 1.2). All participants had normal or corrected-to-normal vision and received small rewards for participating. All participants before inviting to the study were asked about presence of anxiety disorders and depression episodes or taking medications like painkillers or narcoleptics. Only negative response to this questions allow to participate in experiment. The Faculty of Psychology bioethical committee at University of Warsaw and The Maria Grzegorzewska University bioethical committee approved the design of the study and experimental conditions. Participants provide their verbal informed consent to participate in this study. We did not collect written consent due to anonymity of participants assured to them. We did not note any personal data of our participants. Both ethics committees advised us this consent procedure and approve it.

We used three selection criteria. First, all participants were women. Second, all participants were right-handed participants. Third, only participants with an average axiological complexity were recruited for the electroencephalography (EEG) study to reduce inter-individual differences in the sensitivity to words connected with reflective emotions. Axiological complexity is the ability to understand complex concepts by drawing analogies with everyday life. Participants completed an axiological complexity scale which required them to generate referents of concepts such as *loyalty*, by answering questions such as: “How is loyalty manifested in human behavior? Answer by listing examples of loyalty in everyday situations.” Participants included in the study generated a mean 4.2 (*SD* = 1.7) referents per word.

### Linguistic material and its properties

We used a set of 72 Polish words, chosen on the basis of their affective connotations [50]. Words were categorized in terms of the system they engaged (automatic or reflective; 36 words in each origin category) and their emotional valence (negative, neutral or positive; 24 words in each valence category). This allowed us to construct six sets of 12 words for the six possible experimental conditions e.g. negative-automatic. Table 1 shows means (*M*) and standard deviations (*SD*) of the ratings in categories of negative, neutral and positive words as well as automatic and reflective ones. S1 Table, lists all the words with an English translation, their valence and origin categories and frequency of appearance in Polish [52] as well as giving means for valence, arousal and origin [50, 51] and number of letters. Assessments presented in S1 Table were done by 79 women [50] aged from 20 to 25 (*M* = 22.1 *SD* = 1.3). Each word was assessed by from 25 to 28 women. No one from this group participated in EEG study. Origin ratings for stimuli were based on a dichotomous heart vs. mind scale[51]. This scale has been shown to be highly reliable (test-retest reliability in terms of Pearson’s *r* between. 81 and. 90; split-half reliability of. 73 [51]).

**Table 1:** Mean assesmets of Valence, Arousal, Origin and lexical properties of Frequency (Noumber of repetition in Kazojć [52] polish texts base) and Word lenght in case of negative, neutral and positive as well as automatic and reflective words used in the experiment.

		Valence	Arousal	Origin	Frequency	Word length (*N* of letters)
Negative	*M*	2.30	5.73	4.04	1672	9.25
	*SD*	(0.70)	(1.54)	(1.10)	(4594)	(2.33)
Positive	*M*	7.29	4.29	5.45	477	9.88
	*SD*	(0.64)	(1.62)	(1.50)	(703)	(2.94)
Emotional	*M*	4.80	5.01	4.75	1074	9.56
	*SD*	(2.61)	(1.73)	(1.48)	(3307)	(2.64)
Neutral	*M*	5.76	4.91	5.84	375	10.04
	*SD*	(0.90)	(1.26)	(1.26)	(1188)	(1.88)
Automatic neutral	*M*	6.05	5.27	5.02	120	9.33
	*SD*	(0.87)	(1.08)	(0.91)	(116)	(1.23)
Reflective neutral	*M*	5.47	4.55	6.65	629	10.75
	*SD*	(0.87)	(1.37)	(1.02)	(1673)	(2.18)
Automatic emotional	*M*	4.72	5.04	3.88	1578	9.42
	*SD*	(2.96)	(2.20)	(0.94)	(4570)	(2.12)
Reflective emotional	*M*	4.88	4.98	5.61	571	9.71
	*SD*	(2.27)	(1.11)	(1.43)	(963)	(3.11)
Automatic all	*M*	5.16	5.12	4.26	1092	9.39
	*SD*	(2.53)	(1.89)	(1.06)	(3770)	(1.86)
Reflective all	*M*	5.07	4.84	5.96	590	10.06
	*SD*	(1.92)	(1.20)	(1.38)	(1220)	(2.85)
All	*M*	5.12	4.98	5.11	841	9.72
	*SD*	(2.23)	(1.58)	(1.49)	(2794)	(2.41)

On the basis of [50] data set of 78 female psychology students.

We used two-way ANOVA with 3 valence categories (positive/neutral/negative) x 2 origin categories (automatic/reflective) to assess the homogeneity of ratings of the words. We found the following significant moderate or strong effects for assessments of valence, origin, arousal and frequency. Valence categories differ in case of valence assessments (*F*(2, 66) = 341.47, *p* = 1e-35, *η* ^*2*^ = .9), origin assessments (*F*(2, 66) = 23.7, *p* = 2e-8, *η* ^*2*^ = .27), arousal assessments (*F*(2, 66) = 6.29, *p* = .003, *η* ^*2*^ = .16) and frequency estimations (*F*(2, 66) = 3.53, *p* = .03, *η* ^*2*^ = .09). There were no word length effects. Post hoc analysis using simple contrasts showed that all differences were between the negative and positive valence categories. Origin categories differ in case of origin assessments (*F*(1, 66) = 57.87, *p* = 1e-10, *η* ^*2*^ = .33) and frequency estimations (*F*(1, 66) = 4.94, *p* = .03, *η* ^*2*^ = .06). There were no significant valence, arousal and word length effects in case of origin categories.

Pooling the positive and negative categories to give an ‘emotional’ category (as we have done for analysis of origin contrasts in result section) allowed us to analyze the data using a 2 (valence categories (emotional/neutral)) x 2 (origin categories (automatic/reflective)) schema. In this case results of ANOVA revealed expected significant effects of origin assessment between origin categories (*F*(1, 68) = 35.24, *p* = 1e-7, *η* ^*2*^ = .29) and valence assessment between valence categories (*F*(1, 68) = 14.78, *p* = .0003, *η* ^*2*^ = .12). The difference in frequency between valence categories although significant is weak (*F*(1, 68) = 6.2, *p* = .01, *η* ^*2*^ = .08). We did not find any other effects of arousal, frequency or length. What is important from our point of view, there is no significant differences neither in frequency nor in arousal between origin categories.

### Procedure

A standard oddball paradigm was used. Words from pre-defined lists (emotional or neutral) served as the target stimuli. The non-target stimulus, to which the subjects were habituated, was the word *drewno* (‘timber’ in English). Participants were required to read and evaluate words as they were displayed on the screen and respond by a key depending on whether the word was emotional or neutral. They were instructed to avoid evaluating the non-target stimulus (*drewno*) and to withhold the keypress response. Assignment to experimental conditions was random. The sequence of a single trial was as follows: (1) a fixation cross was displayed in the middle of the screen for a random duration in the 0.5–1.5s range; (2) a word was displayed in the middle of the screen for 1s; (3) a blank screen was shown for 2s. Participants were required to respond to stimuli during the 3s period between stimulus onset and the end of the trial.

The experiment consisted of a training session and a measurement session. The training session was included to enable participants to adapt the non-target stimulus; it consisted of 2 target trials and 18 non-target trials presented in random order.

A measurement session consisted of 3 experimental blocks. A single block consisted of six sequences of trials, each sequence presenting target words from one of the 6 sets of words interleaved with the non-target word. The order of sequences within blocks was random. A sequence consisted of randomly selected target stimuli separated by 1 to 6 non-target stimuli, making the average ratio of target to non-target trials 1:3.5. In each experimental condition there were 36 target stimulus presentations (12 words x 3 blocks). To reduce eye-strain there was a pause every 10 to 15 trials. The duration of the pause was regulated by the participant.

### EEG materials

#### Apparatus

Stimuli were controlled by PsychoPy [53] and displayed on a standard PC monitor (LCD display; 17-inch diagonal). A second PC was used for monitoring and recording EEG data. Stimuli and EEG data were synchronized using a custom-made hardware trigger. EEG activity was recorded from 19 electrode sites, Fz, Cz, Pz, Fp1/2, F7/8, F3/4, T3/4, C3/4, T5/6, P3/4, O1/2, referenced to linked earlobes, grounded on the clavicle, with impedances of 5 kΩ or less. The signal was acquired using a Porti7 (TMSI) amplifier with a sampling frequency of 1024 Hz.

#### Offline EEG Signal Processing

The signal was zero-phase high- and low-pass filtered with Butterworth filters (1^st^ order, cut-off frequency = 0.01 Hz; 2^nd^ order with cut-off frequency = 15 Hz respectively). The epochs from -300 ms pre-stimulus to 850 ms post-stimulus were extracted and baseline-corrected (baseline data taken from -300 to -100 ms). After excluding error and artifact trials (e.g. eye blinks or muscle activity) the mean number of trials in the stimulus conditions was as follows: Negative-Automatic, 30.4; Negative-Reflective, 30.0; Positive-Automatic, 29.4; Positive-Reflective 29.1; Neutral-Automatic, 28.8; Neutral-Reflective, 31.0. One-way ANOVA indicated that there was a similar number of trials in all conditions (*F*(5,144) = 0.41, *p*>0.8). In a two-way ANOVA (3 valence x 2 origin) with number of trials as the dependent there was no effect of valence (*F*(2,146) = 0.29, *p*>0.7) or origin (*F*(1,146) = 0.23, *p*>0.6).

We used two methods of data analysis, the standard approach based on analysis of the amplitude of ERP components and independent components analysis (ICA). An ERP component was defined as a deflection in the EEG signal during a specific time range. Four time ranges of interest were selected for further analysis: (1) 200–300 ms; (2) 300–390 ms; (3) 390–590 ms; (4) 590–750 ms. These ranges were selected on the basis of observations of consecutive deflections in the average ERP. Data from all epochs in a given condition were averaged within subjects, and within five regions of interest (ROI): prefrontal (Fp1 and Fp2), frontal (F3, Fz, F4), central (C3, Cz, C4), posterior (P3, Pz, P4) and occipital (O1, O2). Amplitude was measured as the mean amplitude (averaged over the duration of the component) [54] as this is more robust against electrical noise and latency jitter than the maximum amplitude during a given time-range.

We also used ICA. Two components are independent when knowledge about one of them does not provide any information about the value of the other. The EEG signal was decomposed into 19 (equal to available number of EEG channels) independent components (ICs) using the extended ICA algorithm [55]. ICs from different participants were grouped into clusters by using the *K-means* algorithm. Equal weight was given to the similarities in topography and average time course in the distance measure used for clustering. The choice of number of clusters, *K*, was chosen as a compromise such, that for most of the clusters there was only one ICA-component from a given subject, but at the same time there were clusters containing contributions from more than half of the participants. Clusters were only selected for further analysis if they met two criteria: (i) clear dipolar topography, and (ii) contain components from at least half the available sample. It was also ensured, that each participant provides maximum one IC to each of the analyzed clusters.

#### Statistical Methods

For the standard analysis of amplitudes for each time interval we used three-factor repeated measures ANOVA (3 valence x 2 origin x 5 regions of interest). The Greenhouse-Geisser correction for sphericity was applied to the number of degrees of freedom and p-values were adjusted to control for the number of comparisons (Bonferroni correction). A series of paired t-tests was performed to explore significant omnibus effects; Bonferroni-corrected p-values are reported for these tests.

Because ICs are not constrained by the standard peaks and waves of the ERP-waveform we used cluster-based nonparametric tests to analyze them [56]. Comparison of two conditions involved: (i) computation of the test statistic for original labeling of conditions, (ii) estimation of the empirical distribution of the maximum-value cluster-level statistic for the null hypothesis (no difference between the conditions). This was obtained by means of a bootstrap procedure with random shuffling of the condition labels. The test statistic was the sum of the modulus of paired t-values within a cluster of temporally adjacent samples with a modulus t-statistic greater than a threshold *C*. The threshold *C* was defined as the 80^th^ quantile of the distribution of modulus t-values in the pre-stimulus period (-200–0 ms). The cluster-level statistics for the original labeling of the conditions were compared with the (100-5/*n*) quantile of the empirical distributions, where *n* stands for the number of simultaneously tested hypothesis.

## Results

### Behavioral Measures

#### Response Time

Reaction time varied slightly as a function of the emotional valence of the word: *F*(1, 24) = 3.962, *p* = .058, *η* ^*2*^ = .14; reaction times were shorter for negative than positive words: *M*_(Neg)_ = 791 ms (*SEM* = 25) and *M*_(Pos)_ = 827 ms (*SEM* = 25). There was no effects of the origin of the emotion (*F*(1, 24) = .002) and no interaction between origin and valence (*F*(1, 24) = .003) were found.

#### Response Accuracy

Accuracy of responses to the non-target stimulus was subject to a ceiling effect (99%, *SD* = .003), indicating that participants were alert and attentive during the experiment. Trials on which an incorrect response was given were excluded from analyses. Overall the error rate was low in all the experimental conditions (Table 2), but the error rate for a given condition was influenced by task difficulty and to inter-individual differences in assessment of the emotional content of the words. We assessed possible condition effects on error rates using Friedman’s test (null hypothesis of equal error rates for different valence categories, controlling for origin yields: $\chi_{1}^{2}\;=\;15.7$, p = .0004; null hypothesis of equal error rates for different origin categories, controlling for valence: $\chi_{1}^{2}\;=\;8.46$, *p* = .004).

**Table 2:** Assesmet of response error rates—percentage: mean (*SEM*).

	Automatic	Reflective	Total
**Negative**	7.9 (2.1)	8.5 (1.3)	8.3 (1.4)
**Neutral**	16.2 (5.1)	23.4 (4.7)	19.7 (4.6)
**Positive**	17.3 (3.6)	27.4 (5.0)	22.3 (4.0)
**Total**	13.5 (2.0)	19.6 (1.7)	16.6 (1.8)

### Electrophysiological Data

#### Standard Approach

**Fig 1:**
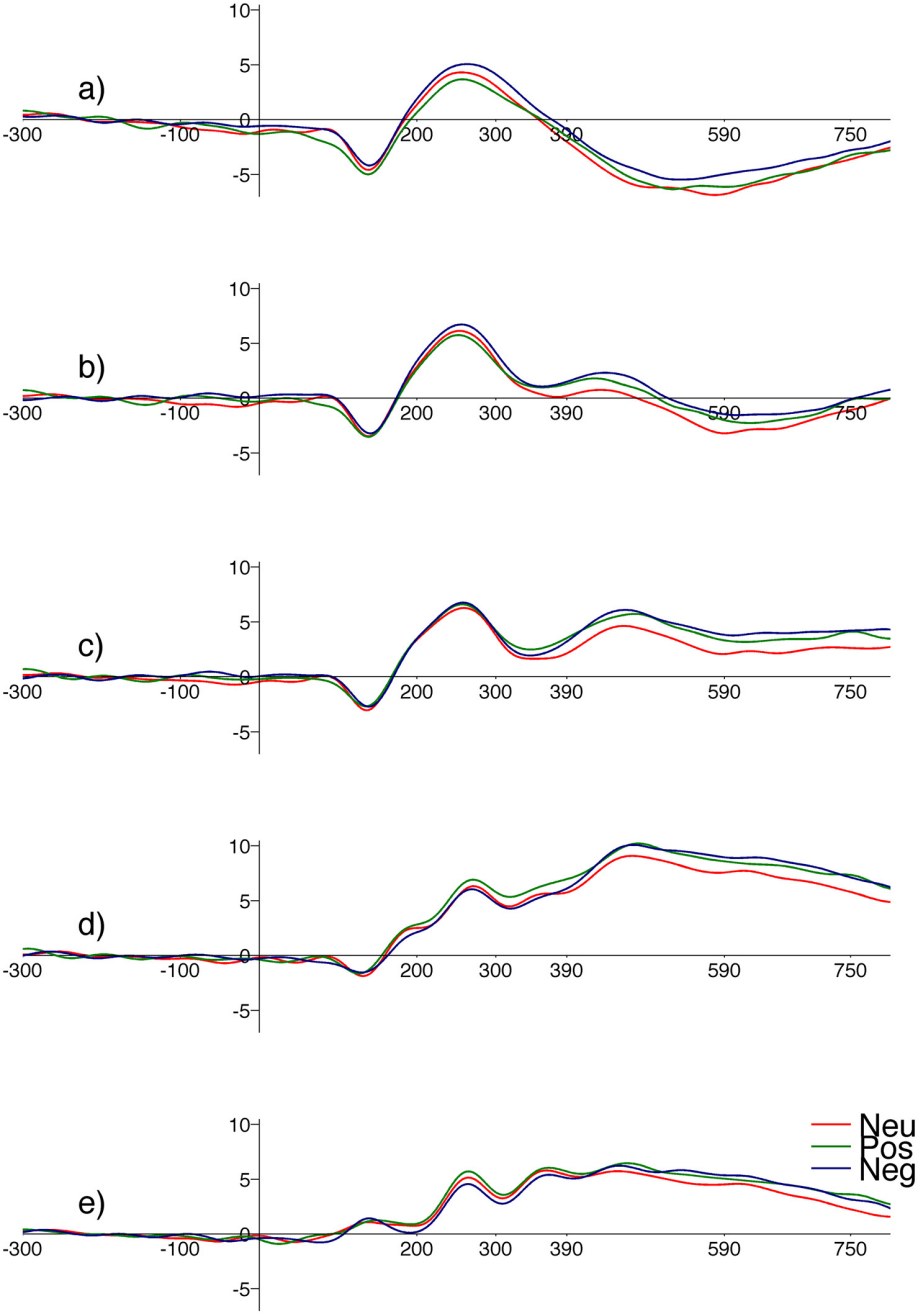
Grand average ERPs with levels of valence overlaid. Red line corresponds to neutral, green to positive and blue to negative valence. The panels are for ROIs: a) PreFrontal, b) Frontal, c) Central, d) Parietal, e) Occipital. Horizontal axis—time in (ms), vertical axis—amplitude in (μV).

Fig 1 illustrates grand average ERPs at different ROIs; the ERPs for the different valence categories have been overlapped. The duration of the intervals analyzed are marked on the horizontal axis. Two effects relating to valence can be observed. In the early period (200–300ms) there was an ordered difference in ERP amplitudes according to valence at the prefrontal and occipital ROIs. In the later period a difference between the ERP to emotional (either positive or negative) and neutral stimuli was visible at the central and parietal ROIs.

**Fig 2:**
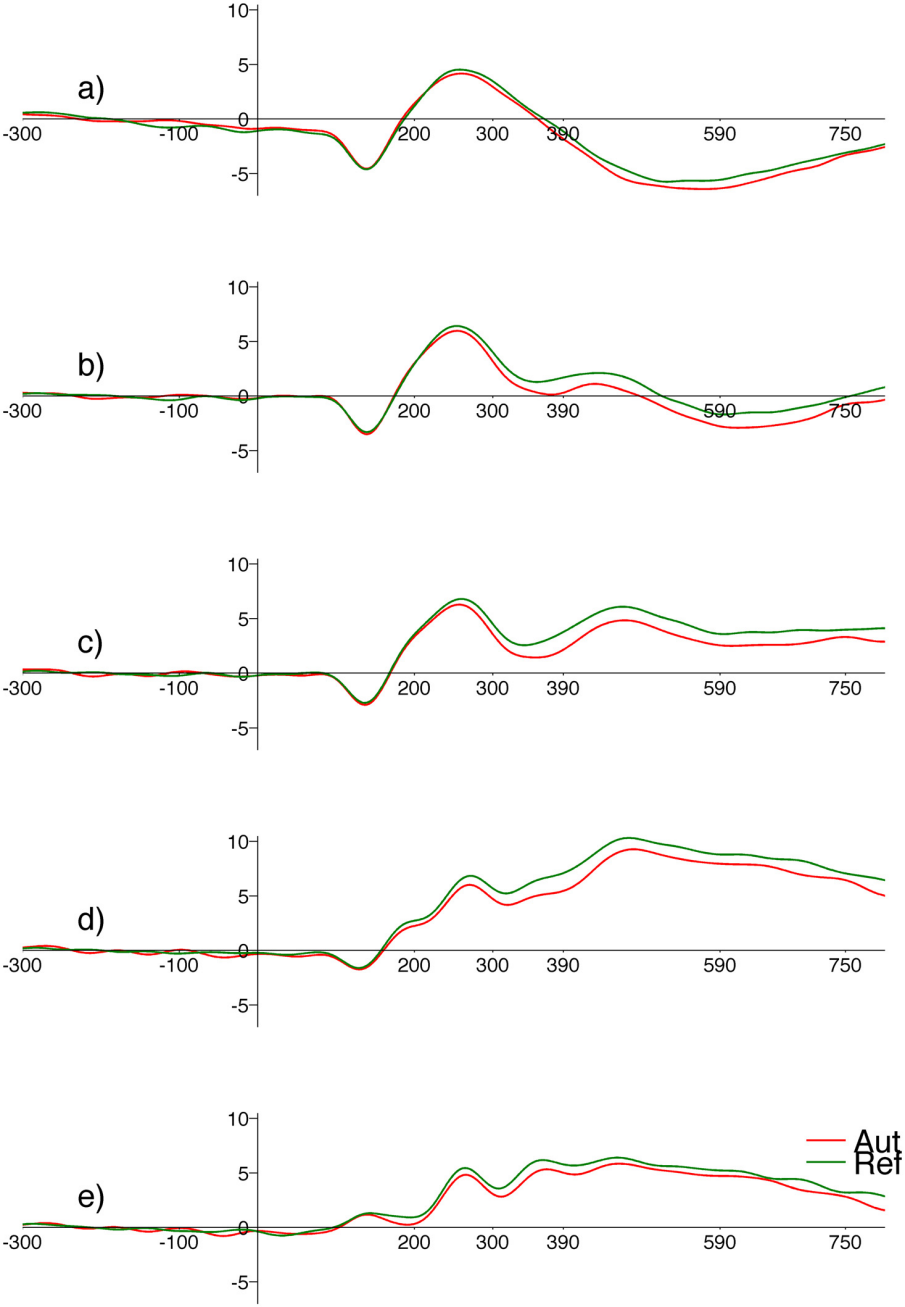
Grand average ERPs with levels of origin overlaid. Red line corresponds to automatic origin, blue line—reflective. The panels are for ROIs: a) PreFrontal, b) Frontal, c) Central, d) Parietal, e) Occipital. Horizontal axis—time in (ms), vertical axis—amplitude in (μV).

Fig 2 illustrates the grand average ERPs at different ROIs; the ERPs for the different origin categories have been overlapped. One can see that after 300ms responses to Reflective stimuli were larger than to Automatic stimuli especially at central and parietal ROIs.

The following sections present the statistical analysis of these effects.

#### Time range 200–300 ms

There were no main effects of valence, origin or ROI. There was a significant interaction between valence and ROI: *F*(8,192) = 6.12, *p* = 2e-6, *η* ^2^ = .2, *ε* = .296. Post-hoc analysis showed that at the prefrontal ROI the amplitudes for negative stimuli (*M* = 4.2, *SEM* = 0.9) were higher than those for positive stimuli (*M* = 2.6, *SEM* = 1); *t*(49) = 3.28, *p* = 0.03); the opposite pattern was observed at the occipital ROI, amplitudes for negative stimuli (*M* = 2.7, *SEM* = 0.5) were lower than those for positive stimuli (*M* = 3.7, *SEM* = 0.4); *t*(49) = -4.1, *p* = 0.002). No other outcomes were statistically reliable. Full results of the contrasts analysis are presented in Table A in S1 File.

#### Time range 300–390 ms

There were significant effects of origin (*F*(1,24) = 11.95, *p* = 0.008, *η* ^2^ = .33, *ε* = 1) and ROI (*F*(4,96) = 8.48, *p* = 3e-5, *η* ^2^ = .26, *ε* = .461). Amplitudes were greater for stimuli connected with reflective (*M* = 3.4, *SEM* = 0.3) than those connected with automatic origin (*M* = 2.4, *SEM* = 0.3); *t*(374) = -6.12, *p* = 2e-9. Post-hoc contrasts showed the effect of origin category was significant in all ROIs except the prefrontal ROIs (details in Table B in S1 File).

#### Time range 390–590 ms

There were significant effects of origin (*F*(1,24) = 18.98, *p* = 0.0008, *η* ^2^ = .44, *ε* = 1), valence (*F*(2,48) = 5.81, *p* = 0.03, *η* ^2^ = .19, *ε* = .89) and ROI (*F*(4,96) = 38.9, *p* = 2e-16, *η* ^2^ = .62, *ε* = .39). Amplitudes were greater for stimuli connected with reflective (*M* = 3.1, *SEM* = 0.4) then for those connected with automatic (*M* = 2.2, *SEM* = 0.4;) origin *t*(374) = -6.31, *p* = 8e-10). Separate contrasts for the ROIs showed that the origin effect applied to the Frontal, Central and Posterior ROIs (Table C in S1 File).

Amplitudes were larger for negative stimuli (*M* = 3.2, *SEM* = 0.4) than neutral stimuli (*M* = 2.0, *SEM* = 0.4); *t*(249) = 6.36, *p* = 3e-9) and also larger for positive stimuli (*M* = 2.8, *SEM* = 0.5) than neutral stimuli (*t*(249) = 3.97, *p* = 0.0003). The effect of valence on amplitude was significant in the Central ROI (Table D in S1 File).

#### Time range 590–750 ms

There were main effects of origin (*F*(1,24) = 9.66, *p* = 0.02, *η* ^2^ = .29, *ε* = 1), valence (*F*(2,48) = 5.59, *p* = 0.03, *η* ^2^ = .19, *ε* = .98) and ROI (*F*(4,96) = 37.02, *p* = 9e-16, *η* ^2^ = .61, *ε* = .35). Amplitudes were larger for stimuli connected with reflective (*M* = 2.0, *SEM* = 0.4) then for those connected with automatic (*M* = 1.2, *SEM* = 0.4); origin *t*(374) = -4.33, *p* = 2e-5). The effect of origin category on amplitude was significant in the Central and Posterior ROIs (Table E in S1 File).

Amplitudes were larger for negative stimuli (*M* = 2.2, *SEM* = 0.4) than neutral stimuli (*M* = 0.9, *SEM* = 0.4; *t*(249) = 5.03, *p* = 3e-6) and also larger for positive stimuli (*M* = 1.7, *SEM* = 0.5) than neutral stimuli (*t*(249) = 3.31, *p* = 0.003). Separate contrasts for the ROIs showed that these valence effects were significant in the Central and Posterior ROIs (Table F in S1 File).

#### Independent Components Analysis

**Fig 3:**
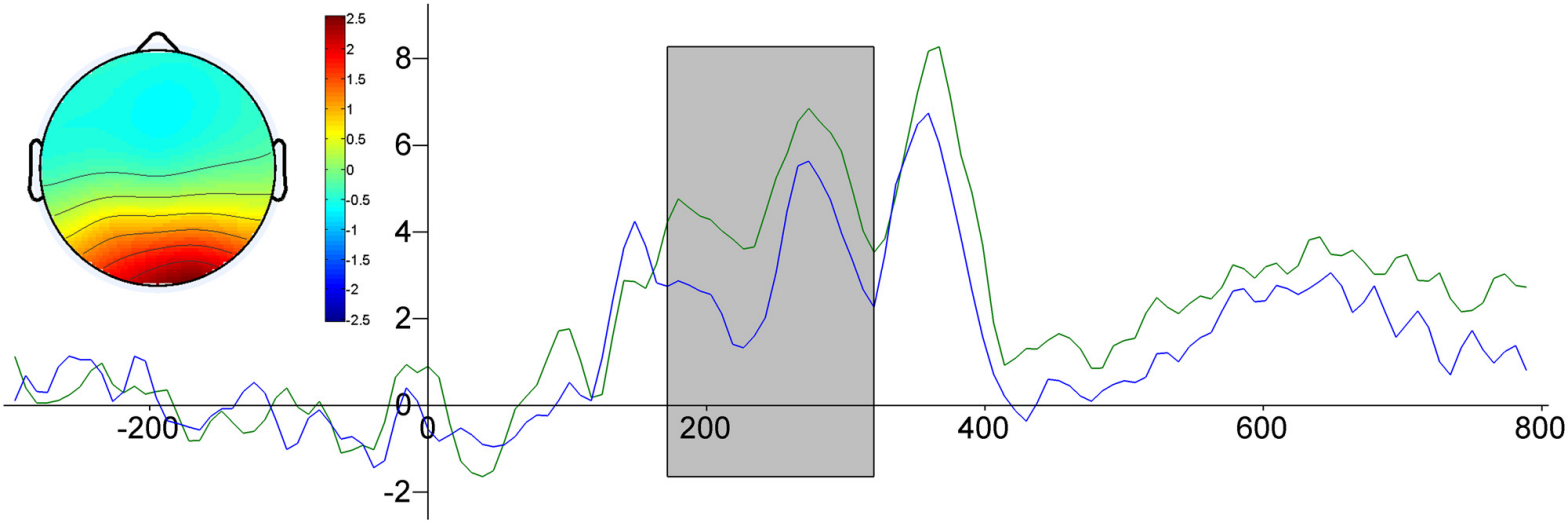
The average ICs. The inset shows the average topography of the cluster. The time course of the response due to different levels of valence: green line—positive, blue line—negative. Horizontal axis time in ms, vertical—amplitude in arbitrary units. Gray rectangle indicates the time intervals with significant differences.

We found one ICA cluster that had properties similar to responses in the early time interval, it is illustrated in Fig 3. The figure shows dipolar occipital topography with the dipole aligned with the central line. As in the standard ERP analysis we found that positive and negative stimuli were associated with significant differences in the amplitudes of the ICs. The significant divergence of the time course of the average ICs in the occipital cluster occurs during period: 180–312ms.

**Fig 4:**
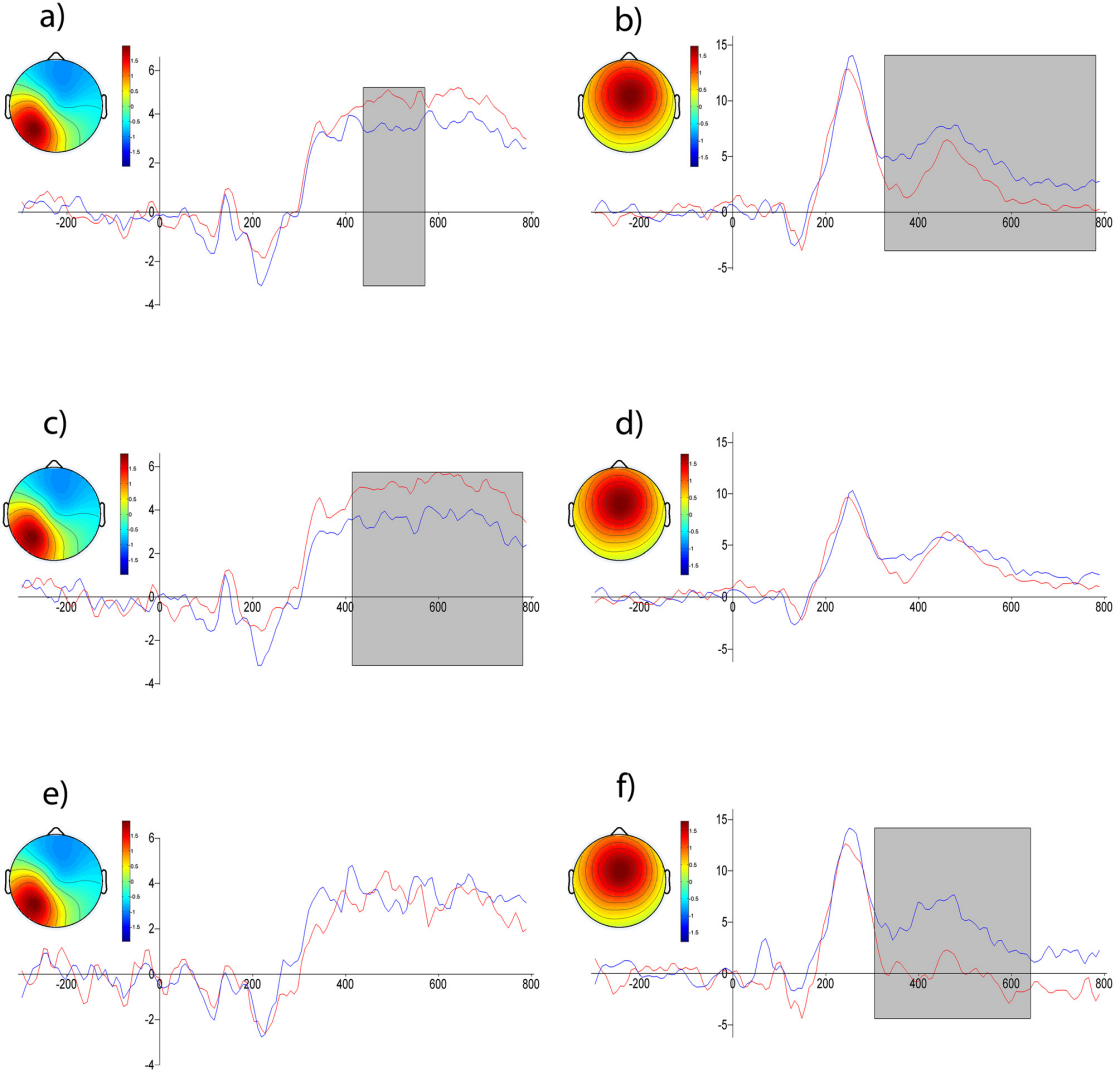
ICs averaged across subjects for two clusters. a) and b) contrast of levels of origin: red line—automatic, blue line—reflective; c) and d) contrast of levels of origin for emotional words only, e) and f) for neutral words only. Horizontal axis—time in ms, vertical—amplitude in arbitrary units. Gray rectangles mark significant differences. Insets depict the cluster topography.

Two clusters had differences in amplitude related to the origin category, one had left-parietal topography (Fig 4a) and the other central topography (Fig 4b). Theses clusters are shown in Fig 4.

During the 437–570 ms period the left-parietal cluster had higher potentials to stimuli connected with automatic origins, whereas the central cluster had higher potentials to stimuli connected with reflective origin during a long period (320–836 ms). Interestingly, when separate contrasts were calculated for neutral and emotional stimuli there were opposite effects of origin category for these two clusters (Fig 4c–4f). In the left-parietal cluster there was a significant difference between the emotional-automatic and emotional-reflective conditions (Fig 4c) but not between the neutral-automatic and neutral-reflective conditions. In the central cluster the opposite pattern was observed, there was a significant difference between the neutral-automatic and neutral-reflective conditions (Fig 4f), but not between the emotional-automatic and emotional-reflective conditions (Fig 4d).

### Modulation of ERP amplitude by stimulus concreteness

After conducting the experiment reported here we found data suggesting that the concreteness of verbal stimuli (emotional and non-emotional words) may influence both EPN and LPC amplitudes during word processing [43, 57, 58]. The amplitude of the LPC was reported to be higher during the reading of abstract words than concrete words. Valence differences in LPC amplitude were only observed for abstract words; more specifically concrete negative words evoked a greater amplitude LPC than positive words [43]. The authors suggested that this effect was due to the activation of mental imagery by concrete stimuli or the activation of semantic context by concrete negative material [43].

We used Pearson’s *r* to assess the possible overlap between the ‘concreteness’ and ‘origin’ dimensions. Word ratings for both dimensions were taken from a study on creation of affective norms which used 4905 Polish words, the methodology was similar to that described in [51], but included new variables, such as the concreteness of words [59]. We found only a weak correlation between origin and concreteness for all words (*r* = .301, *p* < .001). When only the words used in this study were included in the analysis, the correlation was still weak, but in the opposite direction (*r = -*.27, *p* = .02). Furthermore, for the subsets of words used in this study split up according to analyzed categories, we found the following significant strong effects: assessments of concreteness differ between valence categories (*F*(2, 66) = 59.41, *p* < .001, *η* ^2^ = .64) and origin categories (*F*(2, 66) = 57.15, *p* < .001, *η* ^2^ = .46). Neutral (*M* = 4.38, *SD* = 1.03) words were more concrete than positive (*M* = 6.21, *SD* = .70) and negative *(M* = 6.14, *SD* = .94). Automatic (*M* = 4.99, *SD* = 1.25) words were more concrete than reflective (*M* = 6.17, *SD* = .90). Ratings for all words can be found in S1 Table.

To verify our original interpretation of the ICA results we carried out separate assessments of the effect of concreteness on neutral and emotional words. In first case we found that neutral words of automatic origins (*M* = 3.54, *SD* = .18) were more concrete than neutral words of reflective origins (*M* = 5.22, *SD* = .83) (*F*(1, 23) = 49.02, *p* < .001, *η* ^2^ = .49). In second case we found the significant strong effect for only origin categories (*F*(1, 44) = 21.25, *p* < .001, *η* ^2^ = .33). Emotional words of automatic origin (*M* = 5.72, *SD* = .83) were more concrete than emotional words of reflective origin (*M* = 6.64, *SD* = .48).

## Discussion

The major finding of this study is that the processing of the emotional words differs according to which system (AES or RES) is engaged. Words which were assumed to be processed by the AES elicited a smaller amplitude response in the LPC phase than words assumed to be processed by the RES. This difference cannot be attributed to differences in how arousing the words were as both groups of words were similarly arousing. ICA analysis (Fig 4) revealed that this pattern of results was due mainly to neutral words. In the same phase the opposite pattern was observed in another IC localized to the parietal region in the left hemisphere. For this IC component AES-processed emotional words elicited higher amplitude responses than RES-processed emotional words. Taken together these results suggest that there are some differences in the processing of emotional words which can be attributed to differential involvement of the two systems.

### Stimuli

Analysis of the stimuli suggested that the sets of RES- and AES-processed words were relatively homogeneous, but that the valence categories were not. Analysis of affective ratings using Self Assessment Manikins scales revealed that positive and negative words differed in arousal and origins as well as valence. As this was an exploratory study and we were mainly interested in effects associated with the different processing systems (origin categories) we decided to tolerate these effects and take into account the weak to moderate differences in arousal and origin between valence categories.

There were no differences in lexical factors such as word frequency associated with the origin categories, especially when we pooled the positive and negative categories to form a general ‘emotional’ category. Although we were primarily interested in processing system effects the systematic differences between valence groups have to be considered when the results are interpreted.

### Differences in ERPs

The amplitude of an EEG signal is affected by the number of neurons engaged and the degree to which they are synchronized. In the case of perfectly synchronous neurons the amplitude of the response is directly proportional to the number of neurons which are depolarized or hyperpolarized, whilst for completely asynchronous neurons amplitude is proportional to the square root of the number of neurons involved [60]. The greater the number of neurons which respond to a stimulus in a given way, and the more synchronous their activity, the greater the amplitude of the raw EEG signal; in other words the more resources required to process the stimulus, the greater the amplitude of the ERP component associated with these resources. ERP component amplitude therefore can be used as a measure of the intensity of neuronal processes [54, 61]. We investigated two ERP components which have been shown to be connected with processing of emotional words in a variety of tasks [31]. In both cases we found statistically significant effects in standard analyses of ERP amplitudes and ICA.

#### Early Posterior Negativity

Higher EPN amplitudes have been typically interpreted as an index of automatic attention allocation to emotional content [31]. Our results show early differentiation of the response to words according to their emotional valence. The ERP amplitude was higher for positive than for negative words. Standard analyses and ICA produced consistent findings. As Fig 1 shows the ERP waveform at the occipital electrodes was similar to the IC waveform with topography suggesting a dipolar occipital source (Fig 3); in both cases the differences between positive and negative words occurred at a similar latency. These similarities imply that this IC makes a substantial contribution to the EPN. Both standard ERP analysis and ICA indicated that positive words generated higher response amplitudes than negative words.

At first glance this pattern of results could be attributed to differences in the frequency with which the stimuli occur in Polish, but data for words in the neutral category does not fit this interpretation. The mean frequency of neutral words (375) was also different from that of the negative words (1672), yet the neutral and negative words evoked responses of similar amplitude. Facilitated processing of words which appear more frequently cannot account for these data; we suggest that the emotional valence of the word has an influence on processing, which is probably reflected in increased processing costs for positive words.

We suggest that brain is more fluent in processing negative than positive information, and that processing negative stimuli therefore requires less effort. The fact that the error rate for responses was significantly lower for negative stimuli than positive stimuli (Table 2) is consistent with this proposal. Overall our results are similar to those of a study using a subliminal affective priming paradigm with word presentation and registration of evoked potentials [47] which found that the amplitude of responses occurring 250–1000 ms after stimulus presentation was higher for positive subliminally presented words than negative ones.

#### Late Positive Complex

Effects on amplitude at P3 or later time range are thought to be connected mainly to differences in the arousing properties of stimuli [25–27, 39, 46, 47]. Owing to their motivational significance highly arousing stimuli are thought to elicit a more intense neural response [39, 49], which should be reflected in the LPC amplitude.

We found differences in LPC between RES- and AES-processed words even when their arousal level was similar; this poses some problems for the above account of LPC effects. Taking into account the ERP waveform at central locations (Fig 2), the cerebral cortical response to words which were connected with automatic origin was less intense than the response to words which were connected with reflective origin of emotion. This is consistent with the behavioral results (Table 2), namely that the error rate was higher for words assumed to engage the RES. These both differences can be related to a dual-systems account of mind [2, 62] and processing requirements. Words which were connected with automatic origin emotion and are processed by ‘System 1’ (AES in this paper) tend to be those which describe simple, everyday experiences. System 1 processing is effortless. System 2 (RES in this paper) processing (and connected with reflective origin emotions) requires effort and energy is needed to select and execute a response.

We also found that the response to neutral words during the LPC was different from that to both negative and positive words. Emotional words generated ERPs of higher amplitude (Tables D and F in S1 File), presumably because of the greater relevance to subjective goals of the emotionally charged stimuli used in our study (S1 Table). Herbert et al. [63, 64] reported greater amplitude LPCs to self-relevant stimuli (e.g. ‘my happiness’) than self-irrelevant stimuli (e.g. ‘his success’ or ‘her success’). These valence effects are entirely consistent with Citron’s [31] suggestion that the LPC reflects more controlled, explicit processing of emotion, unlike the EPN.

We identified two ICs which contributed to the LPC (Fig 4), differentiated by their topography, sensitivity to the emotional content of stimuli and the direction of their sensitivity to the mental system engaged. In the case of emotional words the central IC had a pronounced peak independent of the system engaged. In case of neutral words, this peak was present only for words assumed to be processed by the RES system. The other component had dipolar left-posterior topography. Reading both emotional and neutral words resulted in a prolonged peak, but in the case of emotional words the amplitude of the peak was greater for AES-processed words than RES-processed words, this may reflect the motivational significance of AES emotional experiences [6]. In the case of neutral words there was no systematic difference in the amplitude of the response associated with the processing system. This complex pattern of effects cannot be attributed to differences in the arousing properties of stimuli, because words in the automatic and reflective categories had similar arousal ratings. This suggests that there are some differences in processing of emotionally charged words which are attributable to the mental system engaged.

### Concreteness of materials used

Correlation analyses in case of affective norms [59] indicate that origin and concreteness share less than 1 percent of their variance and may be considered distinct dimensions. We found also no effects on EPN amplitude that could be attributed to concreteness. Neutral, positive and negative words all evoked responses of similar amplitude independent of stimulus concreteness. We found that more concrete, AES-processed words, tended to evoke an LPC of smaller amplitude than more abstract, RES-processed ones. We also found a valence effect on response in the LPC phases which could be attributed to the difference in concreteness between neutral and emotional words. This interpretation of the results draws on the analysis given by Kanske and Kotz [43].

Additional ICA contrast analyses indicates that the effects on the centrally located IC in the case of neutral words can be interpreted in terms of differences in concreteness, but the effects on the second IC, in the case of emotional words, cannot be interpreted similarly. We found that stimuli which are assumed to engage the AES (more concrete) were associated with higher amplitude responses than stimuli which engage the RES (more abstract). We think that the effects of the engaged system on processing of emotional words are worth further investigation.

### Conclusions

This study is the first, exploratory attempt to show origin of emotion as potentially important factor helpful to understand how emotionally charged words are processed in brain. We propose that emotions can be divided into two main categories, automatic and reflective, according to the system from which they originate. This division reflects the application of duality of mind theories (c.f. [1]) to emotion. Our results suggest that the processing system in which emotions originate shapes the LPC response to emotional words. Unfortunately the data collected in this study do not allow us to discriminate origin and concreteness effects on LPC. The results probably reflect the combined influence of both factors. At least one effect found using ICA, namely that emotional stimuli connected with automatic origin evoke a higher amplitude response than stimuli connected with reflective origin (see Fig 4c) could be firmly attributed to processing system differences; this effect could not be attributed to difference in concreteness or the arousing properties of the stimuli. Further research is needed into effects of processing system on ERPs using stimuli which are homogenous in terms of concreteness.

## Supporting Information

S1 File Additional EEG analysis (Tables A–F in S1 File). (DOCX)

S1 Table List of words used the study. Table presents lexical and affective (valence, arousal and origin ratings by 78 women [50] and concreteness by 25 women and 25 men [59]) properties of words used.
(DOCX)
