# Supplementary material for: Origin of Emotion Effects on ERP Correlates of Emotional Word Processing: The Emotion Duality Approach
Source: PLoS One. 2015 May 8;10(5):e0126129. doi: 10.1371/journal.pone.0126129 (PMC4425658; doi:10.1371/journal.pone.0126129)
Supplement: S1 File — (DOCX) [file pone.0126129.s001.docx]

Table A. Pairwise *t*-test contrasts of amplitude in ROIs for the time interval 200-300 ms and different levels of valence. *P*-table shows the uncorrected *p*-values, the Bonferroni corrected significance level is *alpha*=0.003. Significant differences marked in boldface.

|  | M (SEM) | | | contrast | |
| --- | --- | --- | --- | --- | --- |
| ROI | Neg | Neu | Pos | t(49) | P-table |
| Pre-Frontal | 4.2 (0.9) | 3.3 (0.7) | 2.6 (1) | \|  \| Neg \| Neu \| \| --- \| --- \| --- \| \| Neu \| -1.44 \|  \| \| Pos \| 3.28 \| 1.13 \| | \|  \| Neg \| Neu \| \| --- \| --- \| --- \| \| Neu \| 0.15 \|  \| \| Pos \| 0.002 \| 0.2 \| |
| Frontal | 5.6 (0.6) | 4.9 (0.6) | 4.6 (0.7) | \|  \| Neg \| Neu \| \| --- \| --- \| --- \| \| Neu \| 1.35 \|  \| \| Pos \| 2.81 \| 0.63 \| | \|  \| Neg \| Neu \| \| --- \| --- \| --- \| \| Neu \| 0.18 \|  \| \| Pos \| 0.007 \| 0.5 \| |
| Central | 5.5 (0.5) | 5.1 (0.4) | 5.4 (0.5) | \|  \| Neg \| Neu \| \| --- \| --- \| --- \| \| Neu \| 1.15 \|  \| \| Pos \| 0.64 \| -0.62 \| | \|  \| Neg \| Neu \| \| --- \| --- \| --- \| \| Neu \| 0.3 \|  \| \| Pos \| 0.5 \| 0.5 \| |
| Posterior | 4.4 (0.4) | 4.5 (0.4) | 5.1 (0.4) | \|  \| Neg \| Neu \| \| --- \| --- \| --- \| \| Neu \| -0.47 \|  \| \| Pos \| -2.49 \| -1.88 \| | \|  \| Neg \| Neu \| \| --- \| --- \| --- \| \| Neu \| 0.6 \|  \| \| Pos \| 0.02 \| 0.07 \| |
| Occipital | 2.7 (0.5) | 3.2 (0.5) | 3.7 (0.4) | \|  \| Neg \| Neu \| \| --- \| --- \| --- \| \| Neu \| -2.21 \|  \| \| Pos \| -4.10 \| -1.65 \| | \|  \| Neg \| Neu \| \| --- \| --- \| --- \| \| Neu \| 0.03 \|  \| \| Pos \| 0.0001 \| 0.1 \| |

Table B. Pairwise *t*-test contrasts of amplitude in ROIs for the time interval 300-390 ms and different levels of origin. *P*-table shows the uncorrected *p*-values, the Bonferroni corrected significance level is *alpha*=0.01. Significant differences marked in boldface.

|  | M (SEM) | | contrast | |
| --- | --- | --- | --- | --- |
| ROI | Aut | Ref | t(74) | P-table |
| Pre-Frontal | 4.2 (0.9) | 3.3 (0.7) | -0.74 | 0.5 |
| Frontal | 5.6 (0.6) | 4.9 (0.6) | -2.73 | 0.008 |
| Central | 5.5 (0.5) | 5.1 (0.4) | -3.67 | 0.0004 |
| Posterior | 4.4 (0.4) | 4.5 (0.4) | -4.19 | 7e-5 |
| Occipital | 2.7 (0.5) | 3.2 (0.5) | -3.75 | 0.0003 |

Table C. Pairwise *t*-test contrasts of amplitude in ROIs for the time interval 390-590 ms and different levels of origin. *P*-table shows the uncorrected *p*-values, the Bonferroni corrected significance level is *alpha*=0.01. Significant differences marked in boldface.

|  | M (SEM) | | contrast | |
| --- | --- | --- | --- | --- |
| ROI | Aut | Ref | t(74) | P-table |
| Pre-Frontal | -5.2 (0.9) | -4.6 (0.9) | -1.31 | 0.2 |
| Frontal | -0.4 (0.6) | 0.7 (0.6) | -3.30 | 0.001 |
| Central | 3.7 (0.6) | 5.0 (0.6) | -4.70 | 1e-5 |
| Posterior | 7.9 (0.6) | 9.1 (0.6) | -3.82 | 0.0003 |
| Occipital | 5.0 (0.5) | 5.6 (0.5) | -1.12 | 0.03 |

Table D. Pairwise *t*-test contrasts of amplitude in ROIs for the time interval 390-590 ms and different levels of valence. *P*-table shows the uncorrected *p*-values, the Bonferroni corrected significance level is *alpha*=0.003. Significant differences marked in boldface.

|  | M (SEM) | | | contrast | |
| --- | --- | --- | --- | --- | --- |
| ROI | Neg | Neu | Pos | t(49) | P-table |
| Pre-Frontal | -4 (1) | -5.5 (1) | -5 (1) | \|  \| Neg \| Neu \| \| --- \| --- \| --- \| \| Neu \| 2.35 \|  \| \| Pos \| 1.70 \| -0.65 \| | \|  \| Neg \| Neu \| \| --- \| --- \| --- \| \| Neu \| 0.02 \|  \| \| Pos \| 0.09 \| 0.5 \| |
| Frontal | 0.9 (0.7) | -0.7 (0.7) | 0.3 (0.8) | \|  \| Neg \| Neu \| \| --- \| --- \| --- \| \| Neu \| 3.78 \|  \| \| Pos \| 1.35 \| -1.89 \| | \|  \| Neg \| Neu \| \| --- \| --- \| --- \| \| Neu \| 0.0004 \|  \| \| Pos \| 0.2 \| 0.06 \| |
| Central | 4.9 (0.7) | 3.4 (0.7) | 4.6 (0.7) | \|  \| Neg \| Neu \| \| --- \| --- \| --- \| \| Neu \| 4.58 \|  \| \| Pos \| 0.90 \| -3.26 \| | \|  \| Neg \| Neu \| \| --- \| --- \| --- \| \| Neu \| 3e-5 \|  \| \| Pos \| 0.4 \| 0.002 \| |
| Posterior | 8.8 (0.8) | 7.8 (0.8) | 8.8 (0.8) | \|  \| Neg \| Neu \| \| --- \| --- \| --- \| \| Neu \| 2.92 \|  \| \| Pos \| 0.04 \| -2.73 \| | \|  \| Neg \| Neu \| \| --- \| --- \| --- \| \| Neu \| 0.005 \|  \| \| Pos \| 0.96 \| 0.009 \| |
| Occipital | 5.4 (0.6) | 4.9 (0.6) | 5.5 (0.5) | \|  \| Neg \| Neu \| \| --- \| --- \| --- \| \| Neu \| 1.56 \|  \| \| Pos \| -0.33 \| -1.76 \| | \|  \| Neg \| Neu \| \| --- \| --- \| --- \| \| Neu \| 0.1 \|  \| \| Pos \| 0.74 \| 0.08 \| |

Table E. Pairwise *t*-test contrasts of amplitude in ROIs for the time interval 590-750 ms and different levels of origin. *P*-table shows the uncorrected *p*-values, the Bonferroni corrected significance level is *alpha*=0.01. Significant differences marked in boldface.

|  | M (SEM) | | contrast | |
| --- | --- | --- | --- | --- |
| ROI | Aut | Ref | t(74) | P-table |
| Pre-Frontal | -5.0 (0.8) | -4.5 (0.9) | -0.96 | 0.3 |
| Frontal | -2.4 (0.6) | -1.3 (0.6) | -2.35 | 0.02 |
| Central | 2.6 (0.6) | 3.6 (0.7) | -3.13 | 0.002 |
| Posterior | 7.1 (0.6) | 7.9 (0.7) | -2.52 | 0.01 |
| Occipital | 3.8 (0.4) | 4.2 (0.5) | -1.66 | 0.1 |

Table F. Pairwise *t-*test contrasts of amplitude in ROIs for the time interval 590-750 ms and different levels of valence. *P*-table shows the uncorrected *p*-values, the Bonferroni corrected significance level is *alpha*=0.003. Significant differences marked in boldface.

|  | M (SEM) | | | contrast | |
| --- | --- | --- | --- | --- | --- |
| ROI | Neg | Neu | Pos | t(49) | P-table |
| Pre-Frontal | -4 (1) | -5 (1) | -5 (1) | \|  \| Neg \| Neu \| \| --- \| --- \| --- \| \| Neu \| 1.61 \|  \| \| Pos \| 1.81 \| -0.17 \| | \|  \| Neg \| Neu \| \| --- \| --- \| --- \| \| Neu \| 0.1 \|  \| \| Pos \| 0.07 \| 0.9 \| |
| Frontal | -1.3 (0.7) | -2.5 (0.7) | -1.7 (0.8) | \|  \| Neg \| Neu \| \| --- \| --- \| --- \| \| Neu \| 1.90 \|  \| \| Pos \| 0.85 \| -1.37 \| | \|  \| Neg \| Neu \| \| --- \| --- \| --- \| \| Neu \| 0.06 \|  \| \| Pos \| 0.4 \| 0.2 \| |
| Central | 3.8 (0.8) | 2.2 (0.8) | 3.3 (0.8) | \|  \| Neg \| Neu \| \| --- \| --- \| --- \| \| Neu \| 3.87 \|  \| \| Pos \| 1.10 \| -3.17 \| | \|  \| Neg \| Neu \| \| --- \| --- \| --- \| \| Neu \| 0.0003 \|  \| \| Pos \| 0.3 \| 0.003 \| |
| Posterior | 8 (0.9) | 6.7 (0.8) | 7.7 (0.8) | \|  \| Neg \| Neu \| \| --- \| --- \| --- \| \| Neu \| 3.41 \|  \| \| Pos \| 0.77 \| -3.10 \| | \|  \| Neg \| Neu \| \| --- \| --- \| --- \| \| Neu \| 0.001 \|  \| \| Pos \| 0.4 \| 0.003 \| |
| Occipital | 4.2 (0.6) | 3.5 (0.5) | 4.3 (0.6) | \|  \| Neg \| Neu \| \| --- \| --- \| --- \| \| Neu \| 1.96 \|  \| \| Pos \| -0.07 \| -2.29 \| | \|  \| Neg \| Neu \| \| --- \| --- \| --- \| \| Neu \| 0.05 \|  \| \| Pos \| 0.9 \| 0.03 \| |
