# Supplementary material for: Origin of Emotion Effects on ERP Correlates of Emotional Word Processing: The Emotion Duality Approach
Source: PLoS One. 2015 May 8;10(5):e0126129. doi: 10.1371/journal.pone.0126129 (PMC4425658; doi:10.1371/journal.pone.0126129)
Supplement: S1 Table — Table presents lexical and affective (valence, arousal and origin ratings by 78 women [50] and concreteness by 25 women and 25 men [59]) properties of words used. (DOCX) [file pone.0126129.s002.docx]

S1 Table. List of words used the study. Table presents lexical and affective (valence, arousal and origin ratings by 78 women [50] and concreteness by 25 women and 25 men [59]) properties of words used.

| English word | Polish word | Valence Category | Origin Category | Frequency | Number of letters | Valence | Arousal | Origin | Concreteness |
| --- | --- | --- | --- | --- | --- | --- | --- | --- | --- |
| helplessness | *bezradność* | Neg | A | 297 | 10 | 2.16 | 4.00 | 3.34 | 6.64 |
| disease | *choroba* | Neg | A | 2594 | 7 | 1.65 | 4.17 | 4.19 | 3.72 |
| suffering | *cierpienie* | Neg | A | 1750 | 10 | 1.26 | 5.28 | 2.91 | 6.46 |
| frustration | *frustracja* | Neg | A | 128 | 10 | 2.42 | 7.76 | 3.28 | 6.48 |
| panic | *panika* | Neg | A | 948 | 6 | 1.67 | 8.53 | 1.64 | 6.08 |
| violence | *przemoc* | Neg | A | 551 | 7 | 1.67 | 8.22 | 3.29 | 4.88 |
| horror / terror | *przerażenie* | Neg | A | 2564 | 11 | 1.77 | 7.91 | 2.21 | 6.54 |
| death | *śmierć* | Neg | A | 22744 | 6 | 1.67 | 5.41 | 3.79 | 5.42 |
| threat | *zagrożenie* | Neg | A | 1804 | 10 | 2.22 | 7.87 | 5.20 | 5.32 |
| infection | *zakażenie* | Neg | A | 130 | 9 | 2.06 | 6.03 | 4.60 | 3.58 |
| breakdown | *załamanie* | Neg | A | 338 | 9 | 1.44 | 4.83 | 2.77 | 5.88 |
| enslavement | *zniewolenie* | Neg | A | 48 | 11 | 2.16 | 6.17 | 4.67 | 6.42 |
| selfishness / egoism | *egoizm* | Neg | R | 331 | 6 | 3.68 | 4.72 | 5.81 | 7.14 |
| disgrace | *hańba* | Neg | R | 576 | 5 | 2.03 | 5.59 | 4.34 | 6.76 |
| discredit | *kompromitacja* | Neg | R | 87 | 13 | 1.90 | 6.31 | 4.28 | 6.70 |
| callousness | *nieczułość* | Neg | R | 37 | 10 | 2.19 | 3.38 | 5.72 | 6.70 78 |
| unreliability | *niesłowność* | Neg | R | 4 | 11 | 2.32 | 5.00 | 4.38 | 6.70 |
| indifference | *obojętność* | Neg | R | 1054 | 10 | 3.87 | 2.34 | 6.16 | 6.60 |
| cowardice | *tchórzostwo* | Neg | R | 335 | 11 | 2.63 | 4.93 | 4.20 | 6.74 |
| shame | *wstyd* | Neg | R | 3525 | 5 | 2.34 | 5.70 | 4.33 | 7.08 |
| disappointment | *zawiedzenie* | Neg | R | 2 | 11 | 2.47 | 5.33 | 4.13 | 6.21 |
| abashment | *zawstydzenie* | Neg | R | 83 | 12 | 3.38 | 6.00 | 4.33 | 6.30 |
| embarrassment | *zażenowanie* | Neg | R | 187 | 11 | 2.94 | 6.10 | 4.43 | 6.28 |
| sin | *zgrzeszenie* | Neg | R | 1 | 11 | 3.31 | 6.00 | 3.07 | 6.72 |
| purification | *oczyszczenie* | Pos | A | 181 | 12 | 6.90 | 2.31 | 4.57 | 5.32 |
| relaxation | *odprężenie* | Pos | A | 183 | 10 | 7.87 | 1.56 | 4.04 | 6.08 |
| recapture | *odzyskanie* | Pos | A | 374 | 10 | 6.90 | 5.44 | 5.07 | 5.68 |
| relief / alleviation | *ukojenie* | Pos | A | 334 | 8 | 7.66 | 2.03 | 3.57 | 6.22 |
| relief | *ulga* | Pos | A | 801 | 4 | 7.47 | 2.97 | 3.17 | 6.00 |
| rescue | *uratowanie* | Pos | A | 334 | 10 | 7.88 | 5.83 | 4.53 | 4.90 |
| calm | *uspokojenie* | Pos | A | 280 | 11 | 7.06 | 1.83 | 4.43 | 6.34 |
| release | *uwolnienie* | Pos | A | 563 | 10 | 7.81 | 5.57 | 5.00 | 5.94 |
| rest | *wypoczynek* | Pos | A | 407 | 10 | 8.00 | 2.83 | 4.00 | 5.08 |
| recovery | *wyzdrowienie* | Pos | A | 101 | 12 | 7.81 | 6.20 | 4.57 | 5.4 |
| release | *wyzwolenie* | Pos | A | 406 | 10 | 7.97 | 6.30 | 4.87 | 6.44 |
| relax | *zrelaksowanie* | Pos | A | 0 | 13 | 7.78 | 2.00 | 3.40 | 6.38 |
| pride | *duma* | Pos | R | 1493 | 4 | 7.23 | 5.59 | 4.97 | 7.00 |
| dignity | *godność* | Pos | R | 1132 | 7 | 7.03 | 4.48 | 5.56 | 7.04 |
| honor | *honor* | Pos | R | 3232 | 5 | 6.90 | 4.86 | 5.63 | 7.44 |
| duty | *obowiązkowość* | Pos | R | 15 | 13 | 5.87 | 4.62 | 7.38 | 6.12 |
| courage | *odwaga* | Pos | R | 973 | 6 | 7.23 | 6.63 | 5.29 | 7.16 |
| punctuality | *punktualność* | Pos | R | 90 | 12 | 6.30 | 3.44 | 7.79 | 5.78 |
| self-realization | *samorealizacja* | Pos | R | 5 | 14 | 8.27 | 5.81 | 7.04 | 7.12 |
| satisfaction | *satysfakcja* | Pos | R | 230 | 11 | 8.17 | 5.97 | 5.68 | 6.78 |
| conscientiousness | *sumienność* | Pos | R | 30 | 10 | 6.57 | 3.41 | 7.79 | 6.86 |
| regularity | *systematyczność* | Pos | R | 16 | 15 | 6.37 | 3.44 | 8.21 | 6.38 |
| improvement | *ulepszenie* | Pos | R | 11 | 10 | 7.19 | 5.30 | 6.87 | 5.34 |
| perseverance | *wytrwałość* | Pos | R | 266 | 10 | 6.81 | 4.50 | 7.27 | 6.34 |
| running | *bieganie* | Neutr | A | 90 | 8 | 5.23 | 5.79 | 5.28 | 3.24 |
| going | *chodzenie* | Neutr | A | 387 | 9 | 6.03 | 4.28 | 5.25 | 3.64 |
| cooking | *gotowanie* | Neutr | A | 177 | 9 | 5.94 | 4.97 | 5.66 | 3.68 |
| riding | *jeżdżenie* | Neutr | A | 33 | 9 | 6.65 | 5.83 | 5.69 | 3.72 |
| marching | *maszerowanie* | Neutr | A | 15 | 12 | 5.48 | 4.21 | 5.38 | 3.68 |
| diving | *nurkowanie* | Neutr | A | 84 | 10 | 6.06 | 6.14 | 5.50 | 3.46 |
| baking | *pieczenie* | Neutr | A | 272 | 9 | 4.47 | 4.88 | 5.18 | 3.64 |
| swimming | *pływanie* | Neutr | A | 198 | 8 | 6.60 | 4.88 | 5.29 | 3.34 |
| jumping | *skakanie* | Neutr | A | 58 | 8 | 6.23 | 6.84 | 3.71 | 3.62 |
| dancing | *tańczenie* | Neutr | A | 24 | 9 | 8.00 | 7.03 | 2.63 | 3.76 |
| rowing | *wiosłowanie* | Neutr | A | 54 | 11 | 5.56 | 4.87 | 5.47 | 3.43 |
| sailing | *żeglowanie* | Neutr | A | 47 | 10 | 6.38 | 3.47 | 5.17 | 3.28 |
| silence | *milczenie* | Neutr | R | 5840 | 9 | 6.58 | 4.90 | 5.69 | 5.74 |
| integration | *integrowanie* | Neutr | R | 2 | 12 | 5.35 | 3.24 | 6.38 | 4.34 |
| login | *logowanie* | Neutr | R | 2 | 9 | 4.74 | 1.76 | 5.66 | 4.92 |
| reflection | *myślenie* | Neutr | R | 1129 | 8 | 6.58 | 4.45 | 7.97 | 6.62 |
| calculation | *obliczanie* | Neutr | R | 31 | 10 | 4.84 | 4.24 | 7.75 | 4.38 |
| announcement | *ogłaszanie* | Neutr | R | 7 | 10 | 4.83 | 4.84 | 6.64 | 4.14 |
| programming | *programowanie* | Neutr | R | 46 | 13 | 4.90 | 3.38 | 7.86 | 4.40 |
| convincing | *przekonywanie* | Neutr | R | 53 | 13 | 5.10 | 5.53 | 6.93 | 5.88 |
| transformation | *przekształcanie* | Neutr | R | 39 | 15 | 4.77 | 4.50 | 6.64 | 5.20 |
| formation | *tworzenie* | Neutr | R | 383 | 9 | 7.31 | 7.23 | 4.50 | 5.54 |
| proving | *udowadnianie* | Neutr | R | 10 | 12 | 5.59 | 5.70 | 7.23 | 6.26 |
| implementation | *wdrażanie* | Neutr | R | 9 | 9 | 5.00 | 4.87 | 6.60 | 5.26 |
